# The Mediterranean pyramid: 30 years later, still relevant

**DOI:** 10.1007/s00394-026-04114-4

**Published:** 2026-09-24

**Authors:** Francesco Visioli, Diego Martínez-Urbistondo, Miguel Angel Martínez-González

**Affiliations:** 1 Department of Molecular Medicine, University of Padova, Viale G. Colombo 3, 35121 Padova, Italy; 2 IMDEA- Nutrition, Madrid, Spain; 3 Área de Medicina Vascular, Departamento de Medicina Interna, Clínica Universidad de Navarra, 28027 Madrid, Spain; 4 Fisiopatología de la Obesidad y Nutrición (CIBERObn), Consorcio CIBER, Instituto de Salud Carlos III (ISCIII), Madrid, Spain; 5 Department of Preventive Medicine and Public Health, University of Navarra, Navarra Health Research Institute (IdiSNA), Pamplona, Spain; 6 Department of Nutrition, Harvard T.H. Chan School of Public Health, Boston, MA USA

**Keywords:** Mediterranean diet, Mediterranean diet pyramid, Healthy eating, Olive oil, Plant-based diet, Cardiovascular disease, Liver disease, Chronic kidney disease, Depression

## Abstract

**Purpose:**

The Mediterranean diet, which has its roots in the traditional eating habits of olive-growing regions, is widely recognized for its health benefits.

**Methods:**

Originally described by Ancel Keys and formalized in the 1990s by the Mediterranean Diet Pyramid, this plant-based diet has consistently shown protective effects against cardiovascular disease, type 2 diabetes, various cancers, and cognitive decline. In recent decades, a growing body of evidence — including large randomized controlled trials such as PREDIMED and CORDIOPREV — has further demonstrated the effects on chronic disease prevention and longevity.

**Results:**

In addition to these classic findings, there is growing evidence that the Mediterranean diet is also beneficial in areas such as liver health, particularly steatotic liver disease associated with metabolic disorders, chronic kidney disease, and mental health. Mechanistic studies highlight the role of (poly)phenols, other micronutrients, and fiber in regulating inflammation, oxidative stress, metabolic pathways, and the microbiota. The cultural appeal and palatability of the Mediterranean diet also encourage long-term adherence to the diet.

**Conclusions:**

Overall, this review examines whether the health effects of the Mediterranean diet go beyond the established areas and may represent a holistic strategy for the prevention of a wider range of chronic diseases.

## Introduction

In 1995, a landmark publication by Willett et al. introduced a food pyramid that described the Mediterranean dietary traditions [1]. That publication triggered extensive research into the health effects of the Mediterranean diet, its components, and its implementation as a public health preventive measure [2, 3].

The Mediterranean diet, traditional to the olive-growing regions of the Mediterranean Sea, is one of the healthiest eating habits in the world [4]. Olive oil is an essential component that facilitates the incorporation of vegetables and provides health benefits recognized by a plethora of scientific studies [2, 5, 6]. Well known benefits are increased longevity, reductions in the risk of cardiovascular disease [7–9], type 2 diabetes [10], and some cancers [11] together with the ability to improve cognition [12].

This dietary pattern has a millennia-old tradition [1, 13–15]. Operational definitions of the Mediterranean diet used in epidemiology have not been artificially manipulated to accommodate healthy elements and eliminate those a priori supposed to be unhealthy. On the contrary, these definitions are faithful to the tradition of the geographic area. However, the healthiness of this long-standing dietary model was not duly recognized until a group of prominent scientists specialized in nutrition, public health experts, and scholars of food and culture began to investigate the patterns of the traditional Mediterranean diet in the early 1960s [16]. These efforts, led by world-renowned institutions, culminated in a series of conferences and documents that established the “Pyramid of the Traditional and Healthy Mediterranean Diet,” recognized worldwide. This group of experts spearheaded the International Conference on Mediterranean Diets in 1993, a follow-up conference in 1994, and a series of scientific articles published in special editions of the American Journal of Clinical Nutrition in 1995 and 1997, which sparked international interest in this plant-based and culturally rooted nutritional pattern, changing the course of global nutrition research and public health guidelines [1, 17].

When Ancel Keys first described the Mediterranean diet in the mid-20th century [16], he captured a dietary pattern seemingly endowed with protective properties against chronic disease and premature mortality. Decades later, a consensus conference held in Madrid [13] formalized its principles, setting the foundation for extensive research into its health effects. Observational studies have consistently linked better adherence to the Mediterranean diet with reduced incidence of cardiovascular disease (CVD), cancer, neurodegenerative disorders, and all-cause mortality. Beyond observational epidemiology, large randomized controlled trials have provided first-level evidence [7, 9]. For instance, the PREDIMED randomized trial demonstrated in primary cardiovascular prevention a 31% reduction in major cardiovascular events with Mediterranean diet supplemented with extra virgin olive oil and 28% if supplemented with nuts [18, 19] versus the advice of following a low-fat diet. Subsequently, the CORDIOPREV confirmed in secondary prevention a 26% lower risk of recurrence of cardiovascular events in coronary heart disease patients [20].

### Unparalleled growing accrual of evidence

**Fig. 1:**
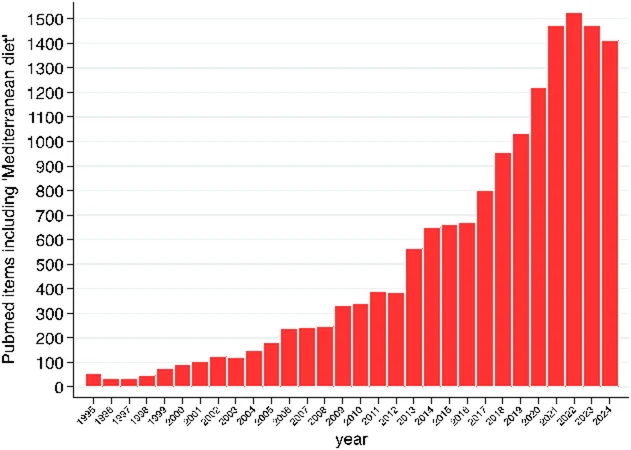
Publications indexed in PubMed including the term “Mediterranean diet” (simple search) from 1995 to 2024. Search conducted on the 26 of March, 2025. For other dietary patterns the total number of publications (without any limit in publication date) were: for “Atlantic diet”, 32 publications in total; for “Nordic diet”, 206 publications in total; for “DASH-Diet”, 2,205 publications in total; for “Mediterranean Diet” 15,667 publications in total

During the last 30 years since the publication of the Pyramid, there has been a global increased interest by investigators in conducting innovative research on the Mediterranean diet and its beneficial health effects (Fig. 1).

The first publication of results from the PREDIMED trial in Spain in 2006 [21] showed clinically meaningful reductions in cardiovascular risk factors. After a few years, this publication was followed by many other studies in different countries assessing cardiometabolic effects of this food pattern. The Mediterranean dietary pattern fitted very well in the emerging paradigm during the first years of the XXI century of the overall food patterns showing an overall healthy eating model, widely regarded as the most effective approach to meeting nutrient requirements. A synergistic effect likely arises from the combination of nutrient-dense foods, which collectively promote favorable changes in intermediate cardiometabolic pathways, as evidenced by randomized controlled trials. Rich in phenolic compounds derived from virgin olive oil, fresh fruits, vegetables, nuts, and legumes, the Mediterranean diet offers significant antioxidant and anti-inflammatory benefits. It also includes moderate consumption of red wine with meals. A defining characteristic of this diet is frugality, marked by minimal intake of processed meats, red meats, and “junk” foods. While each of these dietary characteristics is independently associated with a substantially reduced cardiometabolic risk the cumulative effect of these elements is likely greater than the sum of their individual contributions, underscoring the holistic health benefits of this diet [21].

Thanks to this combination of desirable features, the worldwide interest in studying its effects has been exponentially growing (Fig. 1).

Beyond the well supported benefits on cardiovascular disease, diabetes, cancer and cognitive decline, the Mediterranean diet has been suggested to contribute to the prevention of other diseases in the last two decades. We narratively review the evidence published in the last ten years on outcomes different from cardiovascular disease, diabetes, cancer, cognitive decline, dementia or total mortality. In particular, we review growing evidence on adherence to a Mediterranean-style diet and incidence of hepatic and renal disorders as well as mental health.

### Mediterranean diet and hepatic health

In the realm of liver health, the Mediterranean diet has emerged as a promising strategy against metabolic dysfunction-associated steatotic liver disease (MASLD) which is the most prevalent chronic liver condition worldwide [22]. The rich provision of monounsaturated fatty acids, fiber, and (poly)phenols contribute to insulin sensitivity, lipid metabolism regulation, and consequently to hepatic inflammation reduction [23]. Well-designed observational studies have consistently shown associations between the Mediterranean diet and reduced liver fat content, improved liver enzymes, and decreased fibrosis progression [22–25]. Unlike restrictive diets, the high palatability of the Mediterranean diet facilitates long-term adherence, making it a useful dietary approach for MASLD management [25]. In fact, a meta-analysis of randomized controlled trials, despite finding some heterogeneity, concluded that the Mediterranean diet could alleviate MASLD severity parameters [26]. Subsequently, several other systematic reviews confirmed this conclusion [27–31].

Additionally, a potential protective effect against hepatocellular carcinoma has been suggested, though with some imprecision, by some studies [32–34], further reinforcing the role of this food pattern in liver health.

Beyond liver-specific benefits, the Mediterranean diet plays a crucial role in cardiovascular health, which is particularly relevant given the strong association between MASLD and increased cardiovascular risk. Patients with MASLD frequently present atherosclerosis, hypertension, and insulin resistance, making essential the adoption of a holistic metabolic risk management approach. By addressing both hepatic and cardiovascular health, the Mediterranean diet offers a comprehensive strategy for reducing overall morbidity and mortality in the high-risk population of patients with liver disorders.

Nevertheless, the inclusion of alcohol within the Mediterranean dietary pattern remains controversial in this clinical, i.e. hepatology context [35–37]. Alcohol is a known hepatotoxin and it is identified in clinical guidelines as detrimental to liver health, regardless of the etiology of liver disease [38, 39]. However, when considering competing risks, particularly regarding cardiovascular prevention, moderate alcohol consumption within a Mediterranean pattern could be beneficial in selected individuals [40]. This is a question of utmost epidemiological and precision medicine relevance, which has led to the development of the so-far largest trial of the long-term effects of alcohol cessation versus moderation, namely, the UNATI randomized controlled trial, aimed at evaluating the role of alcohol cessation in the context of the Mediterranean diet [8, 35]. Additionally, a nested sub-study in this trial (UNATI-CUN) will allow for a competitive risk analysis between MASLD and cardiovascular disease, incorporating validated subclinical measurements to assess long-term outcomes.

### Mediterranean diet and kidney health

Chronic kidney disease (CKD) is a global health priority, affecting an estimated 850 million people worldwide [41]. Some observational studies suggested that optimal dietary patterns could be linked to lower risk of CKD [42–44]. Current guidelines [45] supporting the recommendation of the Mediterranean diet at any stage of CKD, also considering its ability to modulate intervening cardiovascular risk factors [46, 47]. Knowledge gaps do exist on its long-term benefits for CKD and only few well-powered randomized controlled trials have evaluated the effect of a Mediterranean diet intervention on kidney function and CKD prevention. The DIRECT trial in Israel (*n* = 318) found that all three energy-reduced weight-loss diets (energy-reduced Mediterranean diet, low-fat diet, and low-carbohydrate diet) exerted similar beneficial effects after two years [48]. The CORDIOPREV trial [20] found significant benefits of the Mediterranean diet in preserving kidney function in survivors of a myocardial infarction [49]. However, the PREDIMED trial assessing participants with type 2 diabetes did not demonstrate additional benefits of the Mediterranean Diet, when compared to a low-fat diet, on incidence of diabetic nephropathy after 6 years, possibly due to similar weight loss in both groups [49].

In recent years, the potential of the Mediterranean diet for general kidney health has gained increasing attention [47]. The emphasis on plant-based proteins, unsaturated fats, and polyphenol-rich foods may help mitigate oxidative stress, inflammation, and endothelial dysfunction, all of which are key drivers of renal impairment [50]. Prospective cohort studies confirmed by randomized clinical trials suggest that higher adherence to the Mediterranean diet is associated with slower CKD progression, reduced albuminuria, and better preservation of glomerular filtration rate [51, 52]. Moreover, its purported [53, 54] low sodium content and high potassium intake contribute to better blood pressure control, a cornerstone in preventing hypertensive nephropathy [55, 56]. Future research should explore the potential role of the Mediterranean diet in modulating gut-kidney axis interactions and fibrosis pathways, offering new perspectives in renal health.

### Mediterranean diet and mental health

Beyond the above-mentioned benefits on cognitive function [12], there is a progressive accrual of convincing evidence supporting a protection by the Mediterranean diet against the occurrence of depressive disorders [57–62]. Concomitant with depression is the production and systemic impact of pro-inflammatory cytokines, including C-reactive protein, interleukin-6, and tumor necrosis factor-α. These mediators have been implicated in the manifestation of depressive symptoms, disruptions in neuroendocrine regulation, increased intestinal permeability, altered monoamine neurotransmission, and impaired neuronal function [56, 63]. It is well-known that they also play a pivotal role in the pathogenesis of cardiometabolic disorders and that the Mediterranean diet is able to counteract these harmful mechanisms [56, 63]. Furthermore, equations for the prediction of cardiovascular disease were also able to predict the future incidence of depression [64]. These facts highlight the plausibility of recent findings showing a broad beneficial influence of the Mediterranean diet on both mental and cardiometabolic health [65]. In a systematic review of different healthy dietary indexes potentially associated with a lower risk of depression, the authors stated that “the most compelling evidence was found for the Mediterranean diet and incident depression, with a combined relative risk estimate of highest vs. lowest adherence category from four longitudinal studies of 0.67 (95% CI 0.55–0.82)” [66] although further evidence on the role of diet on depression risk is needed [67].

In this context, there is a scarcity of long-term trials. The assessments in PREDIMED were basically null, though marginally significant results were found for the group randomly allocated to the Mediterranean diet plus tree nuts [66].

A secondary prevention trial, “PREDI-DEP”, was conducted during the COVID-19 pandemic with substantial challenges for maintaining the contact with patients. It showed, however, significant improvements in the mental health dimensions of the score for measuring quality of life [68].

### Biological plausibility, mechanisms, and further insights

Basic science continues to decode the molecular mechanisms underlying all the aforementioned effects of the Mediterranean diet [69]. Much investigation focused on olive oil, namely the extra virgin one [70, 71], whose (poly)phenols have been studied for decades [72] and can display a European Commission-approved health claim [2, 53, 73]. Indeed, owing to the modest effects of olive oil on cholesterolemia [74, 75], it is important to underscore the need to use high-quality phenol-rich olive oils. Essential fatty acids, which are plentiful in vegetables and marine products [76] have been shown to modulate e.g. inflammation, oxidative stress, and insulin sensitivity [74], while the diet’s low glycemic index [77] and fiber content influence gut microbiota [78] and metabolic homeostasis [79].

In addition to (poly)phenols, plant-based diets such as the Mediterranean one provide important minerals, e.g. potassium and magnesium that improve vascular reactivity and lower the associated inflammatory status [53, 80, 81].

In the Mediterranean basin, except for Maghreb, alcohol is mostly consumed as wine drank – in moderation - with meals [34]. The potential health effects of this pattern of consumption might be different than those attributed to other ways of drinking alcohol, e.g. binge drinking during weekends [34].

Moreover, key components of the Mediterranean diet are increasingly being adopted by other culinary traditions, with olive oil being a prime example of an ingredient that is gaining widespread global acceptance [82]. In addition, the flexibility of the Mediterranean diet pyramid allows it to be adapted to a variety of individual and cultural needs [83, 84], and future trials are expected to provide the evidence base needed to strengthen science-driven public health recommendations.

## Conclusions

**Fig. 2:**
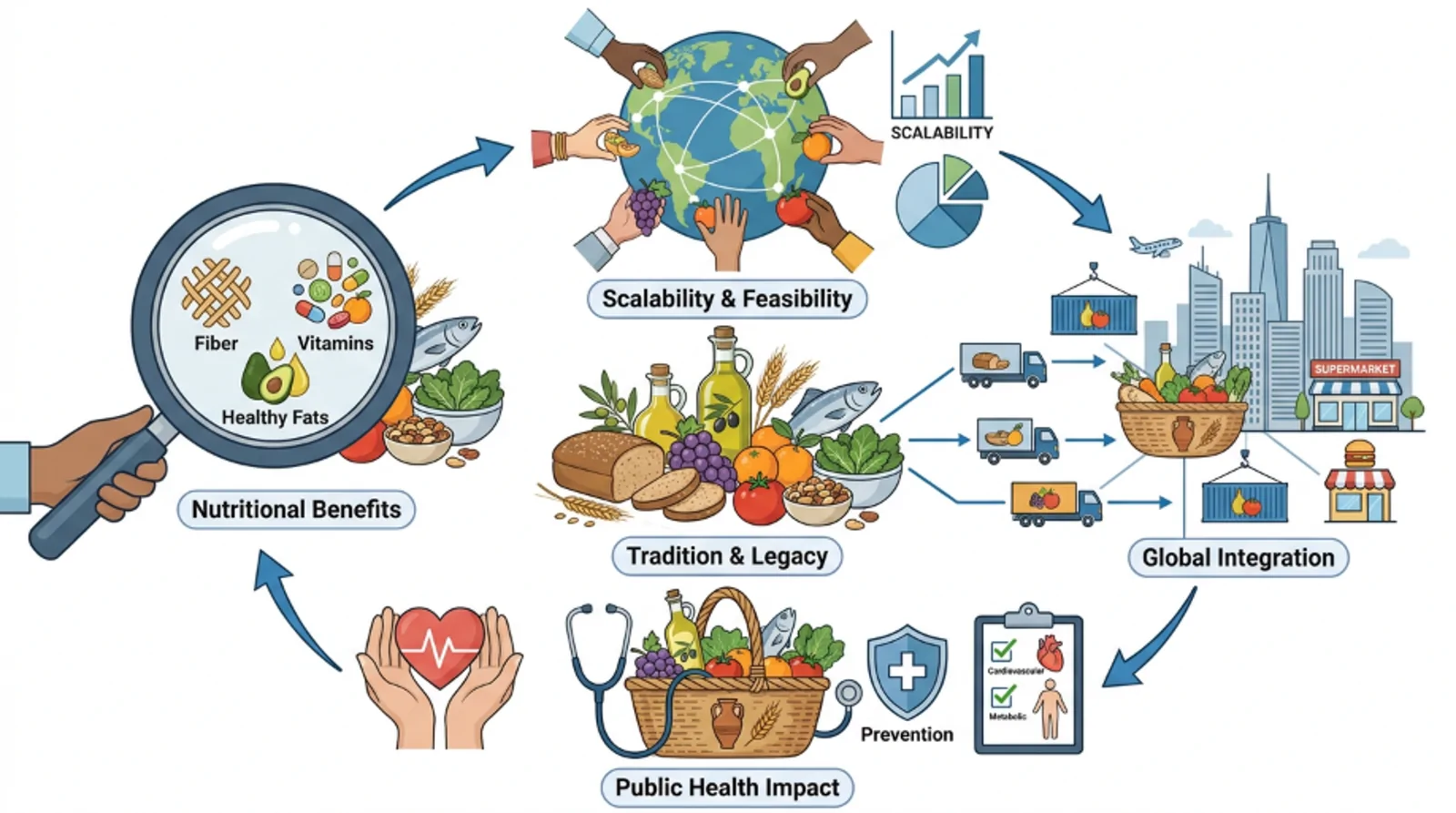
Opportunities and challenges for future worldwide studies. Created with FigureLabs

As we reflect on the past three decades [1], it is clear that while the Mediterranean diet has been extensively studied for its benefits in specific health areas, its potential influence on a broader spectrum of health issues remains largely unexplored. Future research will be crucial in clarifying the mechanisms of action behind the Mediterranean diet and determining its effectiveness in various populations. Of course, from a public health point of view the Mediterranean diet and its components need to be integrated in local culinary traditions (Fig. 2). In addition, future investigations must address the increasingly urgent issues of e.g. climate change (which is impacting production of many fruits and vegetables and their derivatives, including olive oil) [85] and accessibility to healthy diets, which are currently more expensive than unhealthy ones [86] and require some rethinking of investments and subsidies [87].

In conclusion, the Mediterranean diet’s legacy is far from complete. As we look to the future, the integration of this diet into modern food environments will be pivotal in assessing its feasibility as a scalable global health strategy. The ongoing exploration of its benefits will not only enhance our understanding of nutrition but also contribute to the development of effective public health initiatives (Fig. 2).

## Data Availability

None.
